# Management of febrile urinary tract infection among spinal cord injured patients

**DOI:** 10.1186/s12879-016-1484-4

**Published:** 2016-04-16

**Authors:** Aurélien Dinh, Adnène Toumi, Constance Blanc, Alexis Descatha, Frédérique Bouchand, Jérôme Salomon, Thomas Hanslik, Benjamin Bernuz, Pierre Denys, Louis Bernard

**Affiliations:** Infectious Disease Unit, Garches PIFO University Hospital, AP-HP, Versailles Saint Quentin University, Garches, France; Infectious Diseases Unit, University Hospital, Monastir, Tunisia; Pharmacy, University Hospital, AP-HP, Versailles Saint Quentin University, Garches, France; Internal Medicine Unit, University Hospital, AP-HP, Versailles Saint Quentin University, Boulogne-Billancourt, France; Physical Medicine and Rehabilitation Department, University Hospital, AP-HP, Versailles Saint Quentin University, Garches, France; Infectious Disease Unit, Bretonneau University Hospital, Tours, France

**Keywords:** Antibiotic treatment duration, Neurogenic bladder, Urinary tract infection

## Abstract

**Background:**

Urinary tract infection (UTI) among patients with neurogenic bladder is a major problem but its management is not well known. We studied the relationship between antibiotic regimen use and the cure rate of those infections among 112 patients with neurogenic bladder.

**Methods:**

We studied a retrospective cohort of febrile UTI among patients with neurogenic bladder. Drug selection was left to the discretion of the treating physicians, in accordance with current guidelines. Patients were divided into 3 groups according to antibiotic treatment duration (<10 days, between 10 and 15 days, and >15 days). We analysed clinical and microbiogical cure rate one month after the end of antibiotic treatment.

**Results:**

The three groups of patients were similar, especially in terms of drug treatment (equal distribution). The cure rates were not significantly different (71.4 %, 54.2 %, and 57.1 %, respectively; *p =* 0.34). Moreover, there was no difference in cure rate between mono and dual therapy (44 % for monotherapy *vs.* 40 % for dual therapy; *p =* 0.71).

**Conclusion:**

This descriptive study supports the efficacy of antimicrobial treatment duration of less than 10 days and the use of monotherapy to treat febrile UTI among patients with neurogenic bladder. A randomized control trial is required to confirm these data.

## Background

Urinary tract infection (UTI) is one of the most common infectious diseases, especially among patients with neurogenic bladder. These patients are more likely to develop UTI than the general population [1–3]. Fever during UTI suggests the presence of tissue inflammation, which could lead to severe sepsis [4]. Despite voiding practices maintaining low pressure bladder such as intermittent catheterizations, febrile UTI among patients with neurogenic bladder is still the primary cause of morbidity and hospitalization [1, 5].

Guidelines about the duration of antibiotic treatment for acute pyelonephritis are usually 7 to 14 days, and 21 days for complicated forms such as in patients with neurogenic bladder [6]. Shorter antibiotic treatments are considered helpful to reduce the development of antibiotic resistance and reduce toxicity. No recommendations about the duration of antibiotic treatment currently exist for patients with neurogenic bladder.

We aimed to describe cure rates of febrile UTI, according to different antibiotic treatment regimens, in a cohort of patients with neurogenic bladder who were followed in a specialist rehabilitation center.

## Methods

This retrospective study was conducted from January the 1st 2008 to December the 31st 2013 in a University Hospital, with acute care facilities (255 beds, including 43 beds of adult intensive care) and a 108-bed rehabilitation unit (with 40 beds dedicated to spinal cord injured patients). There are about 8400 admissions per year for complete hospitalization. This study was approved by the Saint-Germain-en-Laye ethical (CPP IDF XI) review committee (N° 06070).

### Patients

Hospitalized patients with neurogenic bladder diagnosed with febrile UTI were enrolled in this study if they fulfilled the 3 following criteria: 1) fever > 38 °C; 2) at least one of the following clinical signs lasting less than five days: pyuria, dysuria, frequent urination, urgent urination, perineal or flank pain, clinical autonomic dysreflexia (which is associated with throbbing headaches, profuse sweating, nasal stuffiness, flushing of the skin above the level of the lesion, slow heart rate); 3) a positive urine culture defined as a urine bacterial count ≥10^5^ colony-forming units (CFU)/mL [7] ; 4) management as a UTI by the physician on charge, with relevant and appropriate antimicrobial prescription according to usual guidelines, and absence of other possible diagnosis.

### Methods

Susceptibility to antibiotics was determined using disk diffusion according to the CA-SFM recommendations [8]. For patients with an indwelling urinary catheter, the urine sample was collected from the port of the catheter.

The following information about antibiotic treatment was collected from medical charts using a standard questionnaire:

- Drug treatment
- Duration: patients were divided into three groups according to the duration of antibiotic treatment. Group 1, less than 10 days of treatment; group 2, between 10 and 15 days of treatment; and group 3, more than 15 days of treatment. Duration was the time during which the patient received microbiologically appropriate antibiotic treatment considering the final result of his urine culture.
- Mono or dual therapy: dual therapy was defined as the simultaneous use of two antibiotics effective against the particular pathogens involved, for at least one day during the course of treatment. Only antibiotics that have shown to be effective against febrile UTI according to international guidelines and with proven efficacy based on susceptibility testing were considered for use in dual therapy and treatment duration [6].

The following information was obtained for each case of febrile UTI at diagnosis: clinical data (including type of neurological deficit and voiding practice), bacteriological data, leukocyte count, C-reactive protein level.

### End-point

#### The cure rate

was determined one month after the end of antibiotic treatment from a medical interview [9, 10]. Cure was defined as the complete resolution of symptoms and clinical signs related to UTI without the need for additional or alternative antibiotic therapy. A urine culture was performed at the same time.

#### Failure

was defined as the persistence or progression of symptoms or signs of UTI, or death related to UTI. Patients who could not be evaluated either died due to a condition unrelated to UTI, were lost to follow up, or required another antibiotic treatment during follow up for a concomitant infection outside the urinary tract, were included in the study but not in the outcome analysis. These patients were considered as undetermined.

### Statistical analysis

Data analyses were carried out with the SAS 9.3 software (SAS Institute Inc., Cary, NC, USA). Univariate analyses were carried out with chi^2^ and Fisher exact tests, including all variables known to be associated with UTI (i.e. statistical or clinically significant): sex, age, type of neurological deficit, type of voiding management, positive blood culture [1, 11, 12].

Statistical significance was defined as *p <* 0.05.

## Results

There were 112 cases of febrile UTI among 94 patients with neurogenic bladder. The baseline characteristics of the 94 patients and the outcome of the 112 episodes are listed in Table 1. Of the 94 patients, 83 had a unique episode.

**Table 1:** Characteristics and outcome of 112 febrile urinary tract infection occurring among patients with neurogenic bladder depending on treatment duration

Treatment duration (days)	<10 d	10-15 d	>15 d	Total	*P* value
Number of febrile UTI^a^	28	35	49	112	
Patient characteristics					
Sex (women/men)	12/16	11/24	9/40	32/80	0.2453
Age: mean year (SD)	49.6 (20.1)	46.5 (24.7)	31 (19.7)	38.4 (11.7)	0.1443
Fever: mean °C (SD)	38.6 (0.33)	38.9 (0.76)	39.07 (0.78)	38.8 (0.6)	0.5469
Neurological deficit N (%)					
Paraplegia	18 (64)	20 (57)	27 (55)	65 (58)	0.2604
Tetraplegia	5 (18)	2 (6)	13 (27)	20 (18)	0.5205
Multiple sclerosis	4 (14)	6 (17)	6 (12)	16(14)	0.9299
Brain injury	1 (4)	7 (20)	3 (6)	11(10)	0.1042
Voiding management N (%)					
Intermittent cathererization	14 (50)	13 (37.1)	30 (62)	57 (51)	0.4564
Indwelling catheter	2 (7)	4 (11)	6 (12)	12 (10)	0.9899
Reflex voiding	7 (25)	8 (23)	7 (14)	22 (19)	0.8349
Urinary diversion	2 (7)	6 (17)	3 (6)	11 (10)	
Others	3 (11)	4 (11)	3 (6)	10 (10)	
Biological variables					
Leukocyte count: median/L (SD)	12. 9 (5.5)	13 (6.9)	12 (5.7)	12.6 (6)	0.4426
CRP mean mg/l (SD)	140 (84)	139 (89)	122(90)	121 (84)	0.9953
Positive blood culture N (%)	3 (10)	5 (14)	9 (18)	17 (15)	0.5246
Microorganism sample N (%)					
*E coli*	17 (53)	16 (37.2)	22 (33)	55 (49)	
*Pseudomonas aeruginosa*	6 (18.7)	6 (13.9)	7 (10.6)	19 (13.5)	
*Enterococcus*	4 (12.5)	7 (16.2)	8 (12)	19 (13.5)	
*Klebsiella*	2 (6.25)	4 (9.3)	8 (12)	14 (10)	
*Proteus*	1 (3.12)	3 (7)	4 (6)	8(5.6)	
Others	2 (6.43)	7 (16.4)	17 (26.4))	26 (18.4)	
Antibiotic treatment					
Mean treatment duration (SD)	7.9 (2.3)	13.4 (1.9)	27.3 (9.8)	18.1 (10.7)	0.0250
Mean parenteral treatment duration (median, SD)	8 (8, ±2.2)	6.8 (5, ±5.33)	11.2 (7, ±10.6)	8.2 (6, ±8.3)	
Dual therapy N (%)	17 (61)	18 (52)	34 (70)	69 (62)	0.9669
Antibiotic treatment N (%)					
Third generation cephalosporin	16 (33)	19 (26.3)	32 (32)	67 (30.6)	
Aminoglycosides	12 (25)	18 (25)	21 (21)	51 (23.2)	
Fluoroquinolones	6 (12.5)	14 (19.4)	16 (16)	36 (16.4)	
Penicillin	9 (18.8)	12 (16.7)	14 (14)	35 (16)	
Carbapenem	4 (8.3)	5 (6.9)	11 (11)	20 (9.1)	
Colimycin	1 (2)	2 (2.8)	2 (2)	5 (2.3)	
Cotrimoxazole	0	0	3 (3)	3 (1.4)	
Fosfomycin	0	2 (2.8)	0	2 (1)	
Outcome (1 month)					
Cure N (%)	20 (71.4)	19 (54.2)	28 (57.1)	67 (60.0)	0.3979
Failure N (%) including:	6 (21.6)	10 (28.5)	15 (30.8)	31 (27.5)	
Persistence of symptoms	5 (17.8)	9 (25.7)	12 (24.4)	26 (23.2)	
Death related to UTI	1 (3.5)	1 (3)	3 (6.1)	5 (4.4)	
Patients who could not be evaluated N (%)^b^	2 (7.1)	6 (17.1)	6 (12.2)	14 (12.5)	
Requiring another antibiotic treatment for concomitant infection	0	1	4	5	
Lost to follow up	1	1	1	3	
Death unrelated to UTI	1	4	1	6	
Negative urine culture (D30) N (%)	17 (60.7)	15 (42.8)	21 (42.8)	53 (47.3)	0.635

^a^UTI = urinary tract infection

^b^Patients who could not be evaluated: included death unrelated to UTI (*n =* 6); patients lost to follow-up (*n =* 3), and patients requiring another antibiotic treatment for a concomitant infection (*n =* 5)

The population was mostly male (80/112) with young adults (mean age: 38.4 years old ± 11.7) and paraplegic patients (*n =* 65; 58 %). The most frequent bladder voiding method was intermittent catheterization (*n =* 57; 51 %). During UTI, patients had a mean temperature of 38.8 °C (±0.6 °C) and mean C reactive protein level was 121 mg/L (±84); 15 % (*n =* 17) of patients had positive blood culture.

The main bacteria involved were *E. coli* (*n =* 55; 49 %), *Pseudomonas aeruginosa* (*n =* 19; 13.5 %) and *Enterococcus spp.* (*n =* 19; 13.5 %). Twenty nine (26 %) UTIs were polymicrobial, with four involving 3 microorganisms and 25 involving 2. Mean treatment duration was 18.1 days and dual therapy was prescribed in 62 % (*n =* 69) of cases. Global cure rate was 60 % (*n =* 67) and failure 27.5 % (*n =* 31). Fourteen patients (12.5 %) could not be evaluated.

Eleven patients had recurrent febrile UTI: seven patients had two episodes of UTI, three patients had three, and one patient suffered six. Of these patients, three (27 %) had urinary diversion and three others (27 %) used reflex voiding.

There was no difference between the variables measured in these patients (sex, age, type of neurogenic deficit, voiding practice) and those measured in patients without recurrent UTI who were included in the study. There was no difference from the general population of the study, except for numerous patients with non-stabilized bladder.

The mean duration of antibiotic treatment for these patients was 17 days (1–60).

All kidney examinations and/or abdominal CT scans were normal: no abscess or obstruction. No surgery or derivation had been performed.

The most commonly prescribed antibiotics, considering only appropriate antimicrobial treatment, were third generation cephalosporins (*n =* 67), aminoglycosides (*n =* 51), and fluoroquinolones (*n =* 36).

Considering the three groups of patients, there was no statistically significant difference in sex ratio, type of neurological deficit, positive blood culture, prescribed antibiotics, and mono or dual therapy. There was no significant difference in the cure rate of the three groups (*p =* 0.3979) (assessed by either a Chi^2^ or an exact test). Considering follow up urine culture at day 30, there was no significant difference in bacteriuria rate of the three groups (*p =* 0.635).

Moreover, there was no difference in cure rate between mono and dual therapy (44 % for monotherapy versus 40 % for dual therapy, *p =* 0.071), regardless of the treatment duration.

## Discussion

Our study compared the outcomes of febrile UTI among patients with neurogenic bladder according to different appropriate antimicrobial durations, and mono or dual therapy. We did not find any difference between treatment durations of less than 10 days, 10 to 15 days or more than 15 days. Moreover there was no difference between mono and dual antimicrobial therapies.

There are no well-established guidelines for the optimal antibiotic treatment of febrile UTI in patients with neurogenic bladder. Physicians tend to prescribe antibiotics for a long duration, often with dual therapy, depending on voiding difficulties and on the risk of vesico ureteric reflux. However, long term treatment is considered to be associated with a high risk of toxicity; therefore, this practice may be deleterious and could probably lead to the emergence of multidrug resistant bacteria in patients already frequently exposed to antibiotics [13, 14].

The global low cure rate compared to the rate in usual UTI is often associated with neurogenic bladder [1]. The higher cure rate for group 1, which is the shorter antibiotic treatment group, is possibly linked to the absence of randomization. The fast resolution of symptoms of UTI could have led to shorter antimicrobial treatment.

Considering microbiological data as a cure criterion among a population with neurogenic bladder is under debate because of the frequent asymptomatic bacteriuria in this population [14]. The overall rate of negative urine culture was not statistically different in the three groups.

Our study has several limitations. First it is a non-randomized trial and treatment duration was decided on subjective criteria and depended on the physician in charge and the clinical presentation of the UTI. Several patients (*n =* 3) were lost to follow up, treated with antibiotics for another reason (*n =* 5), or died for another reason (*n =* 6), which is a potential bias. Patients had various neurological deficits and voiding practices, leading to a heterogeneous population, and treatment could have different outcome depending on these bladder characteristics. Also, we considered different microorganisms and antimicrobial treatments. Moreover, changes in antibiotic policy over the 6 years could also have affected the analysis.

Although the statistical strength of this study is limited, it nonetheless contains one of the largest cohorts published to date. These patients were treated at a specialist center.

The treatment duration and the use of mono or dual therapy were decided by the physician because this was a non-interventional trial. However, there was no difference in outcome between the three groups of treatment duration.

Our definition of cure which considers only the absence of clinical symptoms is based on the guideline not to treat asymptomatic bacteriuria in patients with neurogenic bladder [2, 3]. This situation is common in this population and the diagnosis of infection is mainly supported by clinical symptoms. Furthermore, the threshold of bacteriuria to diagnose infection in this population is matter of discussion.

Nevertheless, considering microbiological results of urine culture, we found no difference in the three groups of treatment duration (*p =* 0.635).

Treatment duration is a subject of wide debate. Studies suggest that a short length (5 days) of antibiotic treatment should be encouraged for uncomplicated pyelonephritis [15, 16]. Generally shortening antibiotic treatment duration is a leading goal in public health. It is considered as a tool to fight against bacterial resistance and has several other advantages such as the reduction of side effects and cost. Even if we have not measured these components in our study, we are convinced that short antibiotic treatment duration tend towards these advantages.

The only clinical trial to date about UTI in patients with neurogenic bladder revealed that 14 days of ciprofloxacin treatment is associated with a higher cure rate than only three days of this treatment [5]. Three days may not be enough; however, in our study, we find no difference in the outcome of the three treatment groups. These findings support the trend to use short duration antibiotic treatments of less than 14 days.

The use of dual therapy involving aminoglycosides to treat UTI is controversial [17, 18]. Recommendations usually suggest that aminoglycosides should be used only in cases of severe sepsis or for particular bacteria such as *Pseudomonas aeruginosa* or AmpC-producing *Enterobacteriaceae* [6]. Most of these microorganisms are rare in cases of UTI in the general population but more frequent in patients with neurogenic bladder. Furthermore the high frequency of polymicrobial infection can promote the use of a dual therapy.

Aminoglycosides are potentially highly toxic molecules, and their use should be limited to protect renal function. We suggest that the use of aminoglycosides be avoided for UTI among patients with neurogenic bladder, except for cases of AmpC-producing *Enterobacteriaceae* and severe sepsis.

## Conclusions

As our study found no difference of outcome between long and short antibiotic treatment duration either with mono or dual therapy, this suggests that a long treatment duration and dual antibiotic therapy could be unnecessary for the management of febrile UTI in patients with neurogenic bladder.

According to our data, the management of febrile UTI among patients with neurogenic bladder does not seem to require longer treatment duration than for patients without disability. In our center we recommend an appropriate antimicrobial treatment duration of 8 days for patients with neurogenic bladder.

These promising results should be confirmed by additional data and randomized trials.

## Abbreviations

UTI: urinary tract infection
